# Duffy (Fy), *DARC*, and neutropenia among women from the United States, Europe and the Caribbean

**DOI:** 10.1111/j.1365-2141.2008.07335.x

**Published:** 2008-10

**Authors:** Victor R Grann, Elad Ziv, Cecil K Joseph, Alfred I Neugut, Ying Wei, Judith S Jacobson, Marshall S Horwitz, Natalie Bowman, Kenneth Beckmann, Dawn L Hershman

**Affiliations:** 1 Departments of Medicine, Epidemiology, and Health Policy, Mailman School of Public Health, Columbia UniversityNew York, NY; 2 Herbert Irving Comprehensive Cancer Center, College of Physicians and Surgeons, Columbia University New York, NY; 3 Institute for Human Genetics, Division of General Internal Medicine, Department of Medicine, University of CaliforniaSan Francisco, San Francisco, CA; 4 Department of Biochemistry, Arnold and Marie Schwartz College of Pharmacy and Health Sciences, Long Island UniversityBrooklyn, NY; 5 Department of Epidemiology, Mailman School of Public Health, and Department of Medicine, College of Physicians and Surgeons, Columbia UniversityNew York, NY; 6 Department of Biostatistics, Mailman School of Public Health, Columbia UniversityNew York, NY; 7 Department of Epidemiology, Mailman School of Public Health, Columbia UniversityNew York, NY; 8 Division of Medical Genetics and Medicine, University of WashingtonSeattle, WA; 9 College of Physicians and Surgeons, Columbia UniversityNew York, NY; 10 Children' Hospital Oakland Research InstituteOakland, CA, USA

**Keywords:** ethnic neutropenia, chemokines, *DARC*, African descent, genotype CC

## Abstract

Neutropenia associated with race/ethnicity has essentially been unexplained and, although thought to be benign, may affect therapy for cancer or other illnesses. A recent study linked a single nucleotide polymorphism (SNP) (rs2814778) in the Duffy antigen/receptor chemokine gene (*DARC*) with white blood cell count. We therefore analysed the association of the rs2814778 CC, TC and TT genotypes with absolute neutrophil count (ANC) among asymptomatic women from the Caribbean, Europe and the United States. Among 261 study participants, 33/47 women from Barbados/Trinidad-Tobago, 34/49 from Haiti, 26/37 from Jamaica, and 29/38 US-born black women, but only 4/50 from the Dominican Republic and 0/40 US- or European-born whites (*P* = 0·0001) had the CC genotype. In a linear regression model that included percentage African ancestry, national origin, cytokines, socio-economic factors and the *ELA2* rs57834246 SNP, only the *DARC* rs2814778 genotype and C-reactive protein were associated with ANC (*P* < 0·0001). Women with the CC genotype had lower ANC than other women. Further research is needed on the associations of rs2814778 genotype with neutropenia and treatment delay in the setting of cancer. A better understanding of these associations may help to improve cancer outcomes among individuals of African ancestry.

About 25–40% of blacks in the United States are neutropenic; neutropenia is also prevalent in African and Afro-Caribbean populations (Siebers *et al*, 1989; Bain, 1996; Haddy *et al*, 1999; Bain *et al*, 2000; Hsieh *et al*, 2007; Grann *et al*, 2008). Previous studies comparing Afro-Caribbean and African immigrants to people of African descent born in the United Kingdom found that both groups had similar white blood cell counts (WBC) and absolute neutrophil counts (ANC) and that both groups’ counts were lower than those of UK-born people of European descent. A study of white blood cell response to running a marathon, a reflection of release of neutrophils from bone marrow stores in response to cortisol, showed that runners of African ancestry had a smaller increase in WBC and ANC than white or Asian runners (Bain *et al*, 2000). The WBC response to brief intense exercise, which reflects neutrophil demargination in response to catecholamine release, has also been found to be lower in people of African descent than in those of European descent (Phillips *et al*, 2000). Diet, lifestyle and geographic location appear unlikely to explain the racial differences in leucocyte measures (Bain, 1996), but their biological causes are also not understood.

We recently found that women of African descent with early-stage breast cancer had lower baseline WBC and longer duration of adjuvant chemotherapy than non-Hispanic white women (Hershman *et al*, 2003). Black women were more likely to miss cycles of chemotherapy and had poorer survival than white women. These observations raised questions about whether the prevalence of low WBC varied among ethnic subgroups and how WBC might be related to other biomarkers among women without cancer. Such biomarkers might also help to identify patients with sickle cell disease who would be at increased risk for neutropenia during treatment with hydroxycarbamide (hydroxyurea) (Brawley *et al*, 2008).

The gene that controls expression of the Duffy antigen receptor for chemokines (*DARC*), located on chromosome 1, has long been known to be associated with race. Within the gene, the single nucleotide polymorphism (SNP) most strongly associated with race is *DARC* rs2814778 (Nalls *et al*, 2008). The CC genotype is present among 70–75% of blacks and is rare among whites in the United States. Among individuals with the CC genotype, *DARC* is not expressed (http://www.utdol.com/utd/content/topic.do?topicKey=transfus/11818&view=text). The same genotype was also recently reported to be associated with low WBC (Nalls *et al*, 2008).

We analysed the association of *DARC* rs2814778 genotype with WBC and ANC, as well as other factors thought to be associated with WBC, in an ethnically diverse sample of women without serious illness.

## Patients and methods

### Study design

For this cross-sectional study, we recruited women aged 20–70 years who identified themselves as coming from the United States (US) or one of the Caribbean countries of origin chosen for analysis: Barbados/Trinidad-Tobago, the Dominican Republic, Haiti, and Jamaica. US-born women were eligible if they were either black and had US-born parents, or white. For statistical purposes based on power calculations, we aimed to recruit 40 subjects per ethnic group. Participants represented a convenience sample recruited at Columbia University Medical Center in upper Manhattan or Long Island University in Brooklyn.

Women were screened for enrolment according to place of birth, age at immigration, primary language, current age and medical history. Women who reported a specific illness or disease-related symptoms were excluded from the study.

After obtaining informed consent, we asked the study participants to respond to a questionnaire about demographic and behavioural factors and health history and to provide a blood specimen. A phlebotomist performed venipuncture to obtain a 10 ml blood sample in a yellow-top tube (citrate). Subjects received financial compensation for their participation. Between October 2003 and February 2006, 276 women were enrolled; 13 were excluded because their questionnaire was lost, their blood sample had clotted, or their country of origin was not one of those included in the study; two were excluded because of human immunodeficiency virus infection.

### Laboratory procedures

The Herbert Irving Comprehensive Cancer Center (HICCC) Biomarker Shared Resource and special hematology laboratory at Columbia University Medical Center performed all the analyses except for genotyping. The complete blood counts were determined by Sysmex^TM^ Automated Hematology Analyzer XE-2100. Tumour necrosis factor-alpha (TNF-α) levels were measured with the Human TNF-α/TNFSF1A Immunoassay kit (Quantikine HS; R&D Systems, Minneapolis, MN, USA), granulocyte colony-stimulating factor (G-CSF) levels with the G-CSF Immunoassay kit (Quantikine HS), granulocyte macrophage-colony stimulating factor (GM-CSF) levels using the GM-CSF Immunoassay kit (Quantikine HS), and C-reactive protein (CRP) levels with the High Sensitivity CRP Enzyme Immunoassay Test kit (Life Diagnostics, Inc., West Chester, PA, USA). All immunoassays were performed in duplicate, according to manufacturers’ instructions (Grann *et al*, 2008).

*ELA2* polymorphisms (Horwitz *et al*, 2007) were detected by Sanger dideoxy DNA sequencing of genomic DNA. Polymorphisms indicative of African ancestry, including the rs2814778 SNP, were analyzed using the Sequenom MassARRAY platform and iPLEX Gold chemistry at the Children’s Hospital of Oakland Research Institute (CHORI) Functional Genomics Core (Maglott *et al*, 2007; Wassel Fyr *et al*, 2007; Nalls *et al*, 2008). The MassARRAY platform utilized multiplexed (up to 36-plex) polymerase chain reaction-based target amplification/single-base extension (SBE) coupled with allelic discrimination based on mass differences of SBE products. Detection of mass spectra was achieved with Matrix Assisted Laser Desorption/Ionization Time-of-Flight mass spectrometry.

Individual genetic ancestry was estimated by a maximum likelihood approach (Tang *et al*, 2005).

### Statistical analysis

The frequency distribution of the rs2814778 genotypes (CC, TC, or TT) was analysed by national origin, median ANC, cytokine (TNF-α, G-CSF and CRP) levels, and *ELA2* rs5784246 genotypes (CC, CA, or AA) using chi-square, Fisher’s exact, and Kruskal–Wallis tests. We also analysed the correlation of ANC with African ancestry. The same variables were then included in a linear regression model with log-transformed ANC as the dependent variable. We set the statistical significance criterion as two-sided *P* ≤ 0·05.

## Results

Of the 261 women (Table I) who provided data for the analyses, 47 came from Barbados or Trinidad-Tobago, 50 from the Dominican Republic, 49 from Haiti, 37 from Jamaica, 75 from the United States (38 black and 37 white), and three from Europe (grouped with the US-born whites). The six study groups were similar with respect to age by Kruskal–Wallis test. Of the 183 study participants of Caribbean origin, only 25 (13·7%) were US-born (one whose parents came from Barbados, 14 from the Dominican Republic, four from Haiti and six from Jamaica).

**Table I:** Percentage distribution of Duffy (Fy) blood group system-associated SNPs by country of origin, ANC, cytokines, and *ELA2*rs57834246 allele status.

		CC	TC	TT	
	*N*	(*n* = 121), %	(*n* = 66), %	(*n* = 69), %	*P*-value
Country (race)					
Barbados/Trinidad-Tobago	45	69	20	11	0·0001
Dominican Republic	50	8	40	52	
Haiti	49	75	25	0	
Jamaica	35	63	37	0	
United States (black)	37	73	27	0	
United States or Europe (white)	40	0	5	95	
Overall	256	47	26	27	
Median ANC (×10^9^/l)		2684	4212	3953	<0·0001
% ANC ≤ 1500 × 10^9^/l	4·4	9·3	0	0	0·001
TNF-α (pg/ml)					
Non-detectable	134	48·5	28·4	23·1	0·65
≤233·3	63	47·6	22·2	30·2	
>233·3	59	44·1	23·7	32·2	
G-CSF (ng/ml)					
<0·0259	85	32·9	27·1	40·0	0·002
0·026–0·0381	82	47·6	24·4	28·0	
>0·0381	84	59·5	26·2	14·3	
CRP (μg/ml)					
<1·54	116	44·0	25·9	30·2	0·84
1·55–5·67	66	29·3	22·7	25·8	
>5·67	64	46·9	28·1	25·0	
*ELA2* rs57834246 genotype					
CC	168	36·3	25·6	38·1	0·0001
CA or AA	75	69·3	24·0	6·7	

ANC, absolute neutrophil count; SNPs, single nucleotide polymorphisms; CRP, C-reactive protein; G-CSF, granulocyte colony-stimulating factor; TNF-α, tumour necrosis factor-alpha.

For the total sample of 261 women, the mean and median WBC were 6548 × 10^9^/l and 6200 × 10^9^/l respectively; the mean and median ANC were 3671 × 10^9^/l and 3306 × 10^9^/l.

The *DARC* rs2814778 genotypes were strongly associated with national origin (Table I). No US-born white women and only four Dominican women (8%) had the CC genotype (overall *P* = 0·0001). Women with the CC genotype had lower median ANC (*P* < 0·0001) and higher G-CSF (*P* = 0·002), and were more likely to have the *ELA2* rs57834246 genotype (*P* < 0·0001) than those with the TC and TT genotypes.

**Fig 1:**
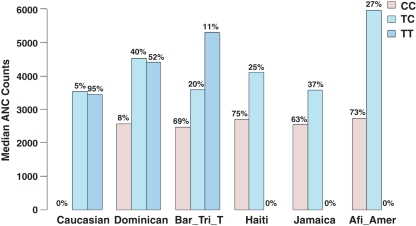
Median ANC counts (x10^9^/l) by *DARC* rs2814778 genotype and ethnic group. The height of each bar represents the median ANC count for women with that genotype in that ethnic group. Above each bar are the percentage distributions of the genotypes in each ethnic group. Bar_Tri_T, Barbados/Trinidad-Tobago; Afi-Amer, African-American.

The median ANC for women with the CC genotype varied little by ethnic group (*P* = 0·91). However, the medians for women with the TC and TT genotypes ranged from 3544 (Caucasian) to 5963 (African American) and from 3444 (Caucasian) to 5304 (Barbadian/Trinidad-Tobago) respectively (Fig 1). Because of small sample sizes, these differences were not statistically significant (*P* = 0·08 for both TC and TT).

In the multivariate model (Table II), only the rs2814778 genotypes and CRP were associated with ANC (*P* = 0·0001).

**Table II:** Linear regression model of factors associated with log-transformed ANC.

	Beta	SD	*P*-value
*DARC* rs2814778 genotype			
CC	Referent		
TC	0·445	0·080	<0·0001
TT	0·476	0·128	<0·0001
% African ancestry			
Below median	Referent		
Above median	0·168	0·168	0·29
Country of origin (race)			
United States (black)	Referent		
Barbados/Trinidad-Tobago	0·023	0·124	0·85
Dominican Republic	0·044	0·159	0·78
Haiti	0·014	0·126	0·91
Jamaica	−0·137	0·156	0·38
United States/Europe (white)	0·108	0·243	0·66
CRP (μg/ml)			
<1·54	Referent		
1·55–5·67	0·076	0·068	0·26
>5·67	0·27	0·069	0·0001
G-CSF (ng/ml)			
<0·0259	Referent		
0·026–0·0381	0·072	0·070	0·31
>0·0381	0·045	0·073	0·54
TNF-α (pg/ml)			
Non-detectable	Referent		
≤233·3	−0·031	0·067	0·64
>233·3	−0·100	0·070	0·16
*ELA2*RS57834246 genotype			
CC	Referent		
CA or AA	−0·038	0·067	0·57
Income			
≤$30 000	Referent		
>$30 000	0·059	0·142	0·68
Education			
≤12th grade	Referent		
>12th grade	−0·105	0·072	0·15
Smoking			
Never	Referent		
Ever	−0·055	0·071	0·44

ANC, absolute neutrophil count; CRP, C-reactive protein; G-CSF, granulocyte colony-stimulating factor; TNF-α, tumour necrosis factor-alpha.

## Discussion

These findings confirm previous observations of an association of the *DARC* rs2814778 SNP with WBC (Nalls *et al*, 2008). This study is, however, the first to show an association between the SNP and low ANC and to identify the association in subjects from the Caribbean as well as from the United States. Our findings also suggest that the low ANC phenotype may be recessive, given the lack of variability in ANC among women with the CC genotype. Women with the TC and TT genotypes had both higher and more variable ANC.

In a recent study of the same sample, clinical neutropenia was found in 12/171 women of African descent and 0/90 white and Dominican women with no reported explanatory medical conditions (Grann *et al*, 2008). Country of origin, *ELA2* rs57834246 genotype, and CRP, although not TNF-α, or G-CSF, were associated with ANC. The addition of *DARC* rs2814778 genotype to the model in the current study eliminated all those associations except for CRP.

The association observed in the current study between higher G-CSF and the *DARC* rs2814778 null homozygous genotype may reflect a feedback mechanism stimulated by low ANC (Semerad *et al*, 2002). G-CSF has been shown both to increase neutrophil production and to accelerate neutrophil entry into the blood (Price *et al*, 1996).

Individuals who lack DARC are resistant to *P. vivax* malaria because the merozoite uses DARC to enter red cells (Miller *et al*, 1976). Individuals who lack DARC on their red blood cells may still express it on their postcapillary venules, where it may interfere with leucocyte trafficking (Peiper *et al*, 1995; Kashiwazaki *et al*, 2003; Nibbs *et al*, 2003).

The mechanism for the association of neutropenia with the *DARC* SNP is unknown. One possibility is that individuals who lack DARC, by which red cells absorb cytokines, are unable to modulate white cell trafficking; hence their neutrophils pool and keep the circulatory ANC low. Such a mechanism might also account for poor mobilization of white cells in response to exercise and stress (Phillips *et al*, 2000; Hsieh *et al*, 2007). One other possibility includes a decrease of bone marrow reserve due to a deficiency of granulocyte–macrophage colony-forming units (Bain *et al*, 2000; Rezvani *et al*, 2001).

Mice that were genetically engineered to lack DARC were unable to bind CXC chemokines, which promote tumorogenesis and angiogenesis (Lentsch, 2006). In a small study (*n* = 75) of human breast cancer pathological samples, high DARC expressors were less likely than low DARC expressors to have extensive lymph node metastases and had lower mortality (Wang *et al*, 2006).

A limitation of this study is that it is based on a convenience sample of women in the ethnic groups of interest. Recruitment took longer for some groups than for others and may have been subject to unknown biases. However, it is difficult to see how bias could account for the association observed between ANC and genotype, neither of which could have been known to the study participants or us prior to the analysis.

Another limitation is that the WBC and ANC were analysed in healthy women. We still know nothing about the association of neutropenia with genotype in the setting of cancer or any other disease. However, the purpose of the study was to learn more about the association between the study markers and ethnicity in the absence of disease.

A third limitation is that the *DARC* rs2814778 genotype may be merely a surrogate marker for another gene that is similarly distributed in the populations studied. However, that SNP is the one most strongly associated with race (Nalls *et al*, 2008). Further studies may consider other candidate genes.

## Conclusion

This study is the first to show an association between the *DARC* rs2814778 CC genotype, common among individuals of African descent, and low ANC. The implications of this association for cancer patients and for those with sickle cell disease remain to be determined. For example, whether the effects of G-CSF as a treatment for chemotherapy-induced neutropenia among cancer patients, and of hydroxycarbamide as prevention for severe pain episodes among sickle cell patients(Steinberg *et al*, 2003), vary by genotype needs to be determined. Further research may help to account for and prevent poor outcomes among persons of African ancestry and lead to interventions that may benefit them and all patients.

## References

[b1] Bain BJ (1996). Ethnic and sex differences in the total and differential white cell count and platelet count. Journal of Clinical Pathology.

[b2] Bain BJ, Phillips D, Thomson K, Richardson D, Gabriel I (2000). Investigation of the effect of marathon running on leucocyte counts of subjects of different ethnic origins: relevance to the aetiology of ethnic neutropenia. British Journal of Haematology.

[b3] Brawley OW, Cornelius LJ, Edwards LR, Gamble VN, Green BL, Inturrisi C, James AH, Laraque D, Mendez M, Montoya CJ, Pollock BH, Robinson L, Scholnik AP, Schori M (2008). National Institutes of Health Consensus Development Conference Statement: hydroxyurea treatment for sickle cell disease. Annals of Internal Medicine.

[b4] Grann VR, Bowman N, Joseph C, Wei Y, Horwitz MS, Jacobson JS, Santella R, Hershman DS (2008). Neutropenia in six ethnic groups from the Caribbean and the United States (in press). Cancer.

[b5] Haddy TB, Rana SR, Castro O (1999). Benign ethnic neutropenia: what is a normal absolute neutrophil count?. Journal of Laboratory and Clinical Medicine.

[b6] Hershman D, Weinberg M, Rosner Z, Alexis K, Tiersten A, Grann VR, Troxel A, Neugut AI (2003). Ethnic neutropenia and treatment delay in African American women undergoing chemotherapy for early-stage breast cancer. Journal of the National Cancer Institute.

[b7] Horwitz MS, Duan Z, Korkmaz B, Lee HH, Mealiffe ME, Salipante SJ (2007). Neutrophil elastase in cyclic and severe congenital neutropenia. Blood.

[b8] Hsieh MM, Everhart JE, Byrd-Holt DD, Tisdale JF, Rodgers GP (2007). Prevalence of neutropenia in the U.S. population: age, sex, smoking status, and ethnic differences. Annals of Internal Medicine.

[b9] Kashiwazaki M, Tanaka T, Kanda H, Ebisuno Y, Izawa D, Fukuma N, Akimitsu N, Sekimizu K, Monden M, Miyasaka M (2003). A high endothelial venule-expressing promiscuous chemokine receptor DARC can bind inflammatory, but not lymphoid, chemokines and is dispensable for lymphocyte homing under physiological conditions. International Immunology.

[b10] Lentsch AB (2006). CXC chemokines and prostate cancer: growth regulators and potential biomarkers. Future Oncology.

[b11] Maglott D, Ostell J, Pruitt KD, Tatusova T (2007). Entrez gene: gene-centered information at NCBI. Nucleic Acids Research.

[b12] Miller LH, Mason SJ, Clyde DF, McGinniss MH (1976). The resistance factor to Plasmodium vivax in blacks. The Duffy-blood-group genotype, FyFy. New England Journal of Medicine.

[b13] Nalls MA, Wilson JG, Patterson NJ, Tandon A, Zmuda JM, Huntsman S, Garcia M, Hu D, Li R, Beamer BA, Patel KV, Akylbekova EL, Files JC, Hardy CL, Buxbaum SG, Taylor HA, Reich D, Harris TB, Ziv E (2008). Admixture mapping of white cell count: genetic locus responsible for lower white blood cell count in the Health ABC and Jackson Heart studies. American Journal of Human Genetics.

[b14] Nibbs R, Graham G, Rot A (2003). Chemokines on the move: control by the chemokine “interceptors” Duffy blood group antigen and D6. Seminars in Immunology.

[b15] Peiper SC, Wang ZX, Neote K, Martin AW, Showell HJ, Conklyn MJ, Ogborne K, Hadley TJ, Lu ZH, Hesselgesser J, Horuk R (1995). The Duffy antigen/receptor for chemokines (DARC) is expressed in endothelial cells of Duffy negative individuals who lack the erythrocyte receptor. Journal of Experimental Medicine.

[b16] Phillips D, Rezvani K, Bain BJ (2000). Exercise induced mobilisation of the marginated granulocyte pool in the investigation of ethnic neutropenia. Journal of Clinical Pathology.

[b17] Price TH, Chatta GS, Dale DC (1996). Effect of recombinant granulocyte colony-stimulating factor on neutrophil kinetics in normal young and elderly humans. Blood.

[b18] Rezvani K, Flanagan AM, Sarma U, Constantinovici N, Bain BJ (2001). Investigation of ethnic neutropenia by assessment of bone marrow colony-forming cells. Acta Haematologica.

[b19] Semerad CL, Liu F, Gregory AD, Stumpf K, Link DC (2002). G-CSF is an essential regulator of neutrophil trafficking from the bone marrow to the blood. Immunity.

[b20] Siebers RW, Carter JM, Wakem PJ, Maling TJ (1989). Racial differences in platelet counts in New Zealand men. New Zealand Medical Journal.

[b21] Steinberg MH, Barton F, Castro O, Pegelow CH, Ballas SK, Kutlar A, Orringer E, Bellevue R, Olivieri N, Eckman J, Varma M, Ramirez G, Adler B, Smith W, Carlos T, Ataga K, DeCastro L, Bigelow C, Saunthararajah Y, Telfer M, Vichinsky E, Claster S, Shurin S, Bridges K, Waclawiw M, Bonds D, Terrin M (2003). Effect of hydroxyurea on mortality and morbidity in adult sickle cell anemia: risks and benefits up to 9 years of treatment. JAMA.

[b22] Tang H, Peng J, Wang P, Risch NJ (2005). Estimation of individual admixture: analytical and study design considerations. Genetic Epidemiology.

[b23] Wang J, Ou ZL, Hou YF, Luo JM, Shen ZZ, Ding J, Shao ZM (2006). Enhanced expression of Duffy antigen receptor for chemokines by breast cancer cells attenuates growth and metastasis potential. Oncogene.

[b24] Wassel Fyr CL, Kanaya AM, Cummings SR, Reich D, Hsueh WC, Reiner AP, Harris TB, Moffett S, Li R, Ding J, Miljkovic-Gacic I, Ziv E (2007). Genetic admixture, adipocytokines, and adiposity in Black Americans: the Health, Aging, and Body Composition study. Human Genetics.

